# Hepatitis B Reactivation Rate and Fate Among Multiple Myeloma Patients Receiving Regimens Containing Lenalidomide and/or Bortezomib

**DOI:** 10.4274/tjh.galenos.2019.2019.0103

**Published:** 2019-11-18

**Authors:** Pınar Ataca Atilla, Merih Yalçıner, Erden Atilla, Ramazan İdilman, Meral Beksaç

**Affiliations:** 1 Ankara University Faculty of Medicine, Department of Hematology, Ankara, Turkey; 2 Ankara University Faculty of Medicine, Department of Internal Medicine, Ankara, Turkey; 3 Ankara University Faculty of Medicine, Department of Gastroenterology, Ankara, Turkey

**Keywords:** Hepatitis B reactivation, Bortezomib, Lenalidomide, Multiple myeloma, Antiviral therapy

## Abstract

**Objective:**

Reactivation of the hepatitis B virus (HBV) refers to an increase in HBV replication in a patient with inactive or resolved HBV. In this retrospective study, our aim is to present and compare HBV reactivation in multiple myeloma (MM) patients who received lenalidomide and/or bortezomib at any time during treatment, evaluate the factors associated with reactivation, and demonstrate the outcome of patients.

**Materials and Methods:**

We evaluated 178 MM patients who received lenalidomide (n=102) and/or bortezomib (n=174) during their treatment schedules. The HBsAg, anti-HBc, anti-HBs, HBeAg, and anti-HBe were detected by chemiluminescence by ARCHITECT lab analyzers using commercially available kits (Abbott, USA). HBV-DNA titers were determined by quantitative PCR. The results were evaluated by IBM SPSS Statistics for Windows, Version 20.0 (IBM Corp., Armonk, NY, USA).

**Results:**

HBV reactivation was diagnosed in 6 patients (3%) after bortezomib and in 8 patients (8%) after bortezomib and lenalidomide. Three of the patients in each group had HBsAg+, HBeAg+, AntiHBeAg-, AntiHBc-, and AntiHBS+ status, whereas 5 patients in the bortezomib- and lenalidomide-treated group and 3 patients in the bortezomib-treated group had HBsAg-, HBeAg-, AntiHBeAg-, AntiHBc-, and AntiHBS+ status prior to treatment. There were no statistical differences observed between HBV reactivation in the bortezomib-treated or bortezomib- and lenalidomide-treated groups in terms of age at diagnosis, sex, International Staging System subtype, frequency of extramedullary disease, dialysis requirement, or receiving of autologous stem cell transplantation. In patients who received antiviral prophylaxis, a higher incidence of HBV reactivation was detected in HBsAg-positive patients compared to HBsAg-negative patients (4/4, 100% vs. 2/7, 29%; p=0.045). The 3-year and 5-year overall survival rates were similar in patients with or without HBV reactivation (83% vs. 84%, 73% vs. 74%, p=0.84).

**Conclusion:**

Close follow-up is recommended for not only HBsAg-positive but also HBsAg-negative patients.

## Abstract (tr)

**Amaç:**

Hepatit B virüs (HBV) reaktivasyonu, HBV enfeksiyonunun inaktifleştiği veya iyileştiği hastalarda virüs replikasyonunun artışıdır. Bu geriye dönük çalışmada amacımız tedavilerinin herhangi bir döneminde lenalidomid ve/veya bortezomib alan multipl myelom (MM) hastalarında HBV reaktivasyonunu göstermek, reaktivasyonla ilişkili faktörleri ve sağkalımlarını değerlendirmektir.

**Gereç ve Yöntemler:**

Tedavileri sırasında lenalidomid (n=102) ve/veya bortezomib (n=174) alan 178 MM hastası değerlendirilmiştir. ARCHITECT lab analiz cihazlarıyla HBsAG, anti-HBc, anti-HBs, HBeAg, anti-HBe piyasada bulunan kitlerle (Abbott, ABD) kemiluminesans yoluyla, HBV-DNA titreleri kuantitative PCR ile tespit edilmiştir. Sonuçların değerlendirilmesinde IBM SPSS 20.0 (IBM Corp., Armonk, NY, ABD) kullanılmıştır.

**Bulgular:**

HBV reaktivasyonu, bortezomib kullanan 6 hastada (%3) ile bortezomib ve lenalidomid alan 8 hastada (%8) tespit edilmiştir. Tedavi öncesi iki gruptan 3 hastada HBsAg+, HBeAg+, AntiHBeAg-, AntiHBc-, ve AntiHBS+ saptanırken, bortezomib ve lenalidomid alan 5 hastada ve sadece bortezomib alan 3 hastada HBsAg-, HBeAg-, AntiHBeAg-, AntiHBc-, ve AntiHBS+ saptanmıştır. Bortezomib veya bortezomib ve lenalidomid ile tedavi edilen gruplar arasında HBV reaktivasyonu ile tanı anındaki yaş, cinsiyet, evre, ekstramedüllar hastalık, diyaliz ihtiyacı veya otolog kök hücre nakil sıklığı arasında istatistiksel olarak fark saptanmamıştır. Antiviral profilaksi alan grupta, HBsAg pozitif olan hastalarda HBsAg negatif olan hastalara göre daha sık HBV reaktivasyonu tespit edilmiştir (4/4, %100 ile 2/7, %29; p=0,045). HBV reaktivasyonu gelişen ve gelişmeyen hastalarda 3-yıllık ve 5 yıllık sağkalımlar benzerdir (%83 ile %84, %73 ile %74, p=0,84).

**Sonuç:**

Sadece HBsAg pozitif hastalar değil HBsAg negatif hastalar da yakından takip edilmelidir.

## Introduction

The hepatitis B virus (HBV) represents a serious health concern worldwide. HBV is intermediately endemic in Turkey, where seropositivity of the hepatitis B surface antigen (HBsAg) has been reported to range between 2% and 7% [1,2]. When there is an increase in HBV replication in a patient with inactive or resolved HBV, this is referred to as reactivation of HBV. Commonly, it occurs in HBsAg-positive cancer patients; HBsAg-negative patients with positive anti-hepatitis B core antibody (anti-HBc) and/or anti-hepatitis B surface antibody (anti-HBs) also carry an increased risk [3,4,5,6]. Cytotoxic chemotherapy, monoclonal antibody treatments, and bone marrow transplantation have been demonstrated as risk factors for HBV reactivation [7,8,9,10]. HBV infection may result in severe hepatic dysfunction and fulminant hepatitis [11,12]. In current treatment guidelines, a prophylactic nucleoside analogue is recommended to be continued for at least 6 months after discontinuation of immunosuppressive therapy [13,14].

Multiple myeloma (MM) is characterized by malignant proliferation of plasma cells. Bortezomib, a proteasome inhibitor that disrupts the cell-signaling pathways, has shown anti-myeloma activity and has been recommended as a standard treatment in patients with newly diagnosed and relapsed MM [15]. Lenalidomide is a potent oral immunomodulatory drug with direct tumoricidal, anti-angiogenic, and immunostimulatory effects [16]. Both bortezomib and lenalidomide show remarkable activity in MM patients with manageable toxicity profiles. There are several case reports and studies on MM showing HBV reactivation under bortezomib treatment [17,18,19], but the literature is scarce regarding HBV reactivation after lenalidomide treatment. In this retrospective study, our aim is to present and compare HBV reactivation in our MM patients who received lenalidomide and/or bortezomib at any time during treatment, evaluate the factors associated with reactivation, and demonstrate the outcome of patients.

## Materials and Methods

We retrospectively included 178 MM patients who were diagnosed between 2002 and 2015 at the Ankara University Faculty of Medicine’s Department of Hematology. Informed consent was obtained from all participants. International Staging System (ISS) scores, counts of hemoglobin and lymphocytes, extramedullary involvement, and plasma cell percentage in bone marrow were recorded at the initiation of chemotherapy. The patients’ data were analyzed via electronic medical records. All patients received lenalidomide and/or bortezomib during their treatment schedules, whether for induction, relapse, or post-induction maintenance.

Hepatitis B surface antigen (HBsAg), hepatitis B core antibody (anti-HBc), hepatitis B surface antibody (anti-HBs), hepatitis B e-antigen (HBeAg), and hepatitis B e-antibody (anti-HBe) were detected by chemiluminescence by ARCHITECT lab analyzers using commercially available kits (Abbott, USA) before each line of chemotherapy. HBV DNA titers were determined by quantitative PCR. Patients with active hepatitis B prior to chemotherapy were excluded from the study. If a patient was HBsAg-positive before chemotherapy or HBsAg-negative but positive for anti-HBc, HBeAg, and/or anti-HBe, a prophylactic antiviral drug was administered during and for at least 6 months after chemotherapy. Hepatitis B serologies were closely monitored in patients who were HBsAg-negative but seropositive for anti-HBc and/or anti-HBs, both before autologous peripheral stem cell transplantation and if liver enzyme abnormality occurred, to determine reactivation. Reactivation was defined as 1) loss of anti-HBs and reoccurrence of HBsAg in HBsAg-negative and/or anti-HBs-positive patients and 2) increase of HBV DNA level by at least a factor of 10 or an absolute count of HBV DNA reaching 1x10^9^ copies/mL. Antiviral treatment was initiated as soon as reactivation was detected. None of the patients had received hepatitis B vaccinations.

### Statistical Analysis

The results were evaluated by IBM SPSS Statistics for Windows, Version 20.0 (IBM Corp., Armonk, NY, USA). All numerical values are given as medians with distribution ranges. We used the Pearson chi-square test or the Fisher exact test to compare categorical variables. The Kaplan-Meier method was used for survival curves. In evaluating the results, p<0.05 was considered statistically significant.

## Results

The median age of the 178 MM patients was 62 (range: 34-86). The baseline characteristics of the study population are summarized in Table 1. At diagnosis, the mean lymphocyte count and hemoglobin concentration were respectively 1936/mL (range: 200-13200) and 11.5 g/dL (range: 7-16). Subjects received a median of 3 lines of treatment (range: 1-7). First-line regimens were as follows: for 80 patients (45%), bortezomib + cyclophosphamide + dexamethasone (VCD); 40 patients (22%), vincristine + doxorubicin + dexamethasone (VAD); 21 patients (12%), cyclophosphamide + dexamethasone (Cy-Dex); 15 patients (8%), bortezomib + dexamethasone (Vel-Dex); 12 patients (7%), bortezomib + melphalan + prednisolone (VMP); 7 patients (4%), melphalan + prednisolone + thalidomide (MPT); and 3 patients (2%), lenalidomide + dexamethasone (Len-Dex). In total, 124 patients (70%) were treated with high-dose chemotherapy and underwent autologous hematopoietic stem cell transplantation (auto-HSCT). During the treatment period, 102 patients (57%) received 25 mg/day lenalidomide with dexamethasone or 10 mg/day lenalidomide as a single agent; 174 patients (98%) received 1.3 mg/m^2^ bortezomib in combination with dexamethasone, cyclophosphamide plus dexamethasone, lenalidomide plus dexamethasone, or melphalan plus prednisone. Bortezomib and lenalidomide were administered to 98 patients (55%). Disease relapse was detected in 122 patients (69%). During follow-up, 41 patients (23%) had progressive disease and 37 patients (21%) died. Herpes virus reactivation (herpes zoster) was detected in 15 patients (8%), 2 of whom received lenalidomide and bortezomib.

Among all subjects, HBsAg was positive in 4 patients (2%) at diagnosis. Among HBsAg-positive patients, 3 patients had HBV DNA levels of >1000 IU/mL. For prophylaxis, patients received either 100 mg of lamivudine (n=2) or 245 mg of tenofovir (n=2) daily, which continued for 6 months after termination of treatment for MM, except in 1 patient who died of infection in the second month of chemotherapy. Among HBsAg-negative patients who were positive for anti-HBc, anti-HBe, or HBeAg (n=7), 6 patients received 100 mg/daily lamivudine, and 1 patient had entecavir at 0.5 mg/daily for prophylaxis that was prolonged for 6 months after treatment of MM. All HBsAg-negative patients had HBV DNA levels of <500 IU/mL. No significant differences were observed in sex, age at diagnosis, ISS stage, subtype, frequency of extramedullary disease, or dialysis requirements between HBsAg-positive and HBsAg-negative patients.

**Figure 1:**
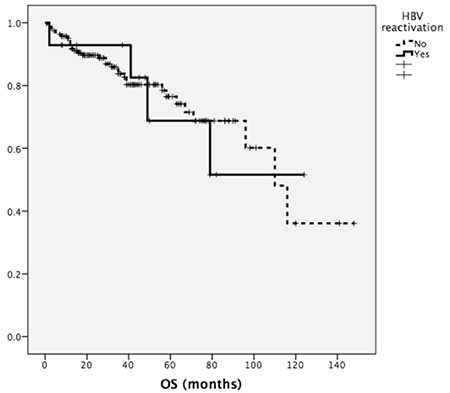
Comparison of overall survival in patients with or without hepatitis B virus reactivation (p=0.84). OS: Overall survival, HBV: hepatitis B virus.

Hepatitis B reactivation was observed in 14 patients (8%). The patients’ HBV and prophylaxis statuses at diagnosis are summarized in Table 2. The median time from diagnosis to hepatitis B reactivation was 32 months (range: 2-78). Of 174 bortezomib-treated patients, 6 had HBV reactivation (3%). HBV reactivation was detected in 8 patients out of the 98 patients who received lenalidomide and bortezomib (8%). Reactivation developed in 4 patients (100%) who were HBsAg-seropositive at diagnosis, while 10 patients (6%) were initially HBsAg-negative. HBsAg-positive patients who received prophylaxis had significantly higher incidence of hepatitis B reactivation than HBsAg-negative patients (4/4, 100% vs. 2/7, 29%; p=0.045). The 3-year and 5-year overall survival (OS) was similar in patients with and without HBV reactivation (83% vs. 84%, 73% vs. 74%, p=0.84) (Figure 1). Details of patients with HBV reactivation are given in Tables 3 and 4. Patient number 5 in Table 4 had HBV reactivation under lamivudine prophylaxis and died because of bacterial infection following 2 months of chemotherapy. Chemotherapies were suspended until liver function tests and HBV DNA levels were decreased.

Baseline characteristics including MM subtype, extramedullary disease, median age, sex, ISS, incidence of herpes infection, and auto-HSCT did not differ between the bortezomib- and lenalidomide-treated vs. bortezomib-treated groups that had HBV reactivation. Lenalidomide treatment was interrupted in 4 (50%) of the patients due to progression of disease. Except for 1 patient, all patients underwent autologous stem cell transplantation (ASCT), and 1 patient who received a second ASCT for a secondary refractory disease had progression to cirrhosis following high-dose melphalan. After treatment with tenofovir, HBV DNA titers decreased in all patients and became undetectable in 4 of the 8 patients. In patients treated with only bortezomib, all patients received dexamethasone, and 4 of 6 patients underwent ASCT. Progression of disease after bortezomib was detected in 2 patients. Among these 6 patients, 4 patients were treated with tenofovir (2 achieved HBV DNA negativity), and the other 2 were treated with lamivudine. The response could not be evaluated for patient number 5, because she died of infection within 2 months of the initiation of chemotherapy (Tables 3 and 4).

## Discussion

Generally, HBV reactivation has been documented in HBsAg-positive cancer patients [20]. In one study, the rate of HBsAg seropositivity in MM cases was higher than in patients with acute leukemia [21]. Antiviral prophylaxis is the critical step in managing HBsAg-positive patients undergoing systemic chemotherapy [13,22]. Clinical studies showed a reduction of HBV activation rate, severity of hepatitis, and mortality with prophylaxis [23,24]. The American Gastroenterological Association suggests antiviral drugs with high barriers to resistance rather than lamivudine for at least 6 months in high-risk patients [14]. Previously, in our experience, because HBV reactivation in a lamivudine-untreated group occurred 12 months after the individual’s chemotherapy had been discontinued, lamivudine prophylaxis was maintained for a year following discontinuation of any chemotherapy [25,26]. The choice of lamivudine or a shorter duration of prophylaxis might have caused the HBV reactivation that occurred in all HBsAg-positive patients who received prophylaxis in this cohort. One patient with HBV reactivation died under lamivudine prophylaxis within 2 months of chemotherapy. Recent data have shown HBV reactivation in HBsAg-negative lymphoma patients who received rituximab plus steroid combination chemotherapy [3,4,27]. Lee et al. [28] demonstrated HBV reactivation in 5.2% of 230 MM patients. All of these patients had HBsAg-negative/anti-HBc-positive serology. Similarly, we found that the incidence of HBV reactivation in HBsAg-negative patients was 6%. The preferred prophylaxis was lamivudine in HBsAg-negative patients.

This is the first study of the recently developed agents lenalidomide and bortezomib in MM, and we observed an incidence of HBV reactivation of 8%. HBV reactivation after bortezomib was described in previous case reports [17,18,19]. Mya et al. [29] found an incidence of HBV reactivation of 5.5% in 273 MM patients after bortezomib and dexamethasone salvage therapy; one of the HBV reactivation cases was HBsAg negative initially. Li et al. [30] conducted one of the largest retrospective studies of HBV reactivation in patients who received regimens containing bortezomib. HBV reactivation was observed in 6 HBsAg-positive and 2 HBsAg-negative cases from a total of 139 patients. OS and progression-free survival were shorter in HBsAg-positive MM patients compared to HBsAg-negative patients (p<0.01) [30]. We did not detect any survival advantage in HBsAg-negative patients in our study. Bortezomib dysregulated the cell-mediated immunity that played an important role in the suppression of varicella zoster virus reactivation [31]. HBV is another DNA virus that remains dormant in human hosts. Bortezomib may promote HBV reactivation by altering the number and functions of CD8 T cells and CD56 NK cells [29]. In addition, MM itself causes immunodeficiency that involves various parts of the immune system, including B, dendritic, T, and NK cell dysfunction. HBV reactivation after lenalidomide has not been reported previously in the literature. König et al. [32] reported 10 varicella zoster virus or other complicated VSC/herpes simplex virus infections from 93 MM patients who received lenalidomide-based chemotherapy, which may have resulted from the immunomodulation effects of lenalidomide. Since the patients in our study were heavily pretreated, and there was no control group assigned for patients not receiving either bortezomib or lenalidomide, it is not clear whether the HBV reactivation was driven by bortezomib and/or lenalidomide. Multiple lines of treatment may cause severe immunosuppression that results in an increased risk of HBV reactivation [33].

Auto-HSCT was shown to be a risk factor for HBV reactivation in several reports. Uhm et al. [34] retrospectively analyzed changes in HBV serology prior to and following auto-HSCT and concluded that 6 of 129 HBsAg-negative MM patients became HBsAg-positive, possibly related to dysfunction of humoral immunity. Lee et al. [28] determined auto-HSCT to be an independent risk factor (p=0.025) for HBV reactivation and suggested that regular monitoring should be considered in patients who underwent auto-HSCT [28]. However, we did not find a significant correlation between HBV reactivation and auto-HSCT.

HBV reactivation may be variable, from mildly clinical to hepatic failure. Development of fatal hepatitis following HBV reactivation was reported in CD20-positive lymphoma patients who received rituximab and steroid combination treatment [7,27]. Yoshida et al. [35] described HBV reactivation in 2 HBsAg-seronegative MM patients resulting in liver damage. Similarly, one of our heavily pretreated patients with HBV reactivation had disease with liver damage progressing to cirrhosis following a second ASCT treatment.

## Conclusion

We found that the incidence of HBV reactivation was notable in patients who received lenalidomide- and/or bortezomib-based chemotherapy. Most of the patients were heavily pretreated, which might have caused immune deficiencies. HBV reactivation was diagnosed in both HBsAg-positive and HBsAg-negative patients. This finding suggests a close follow-up strategy in HBsAg-positive patients as well as HBsAg-negative but anti-HBc-, HBeAg-, or anti-HBe-positive MM patients, plus early initiation of active antiviral therapy.

## Figures and Tables

**Table 1:**
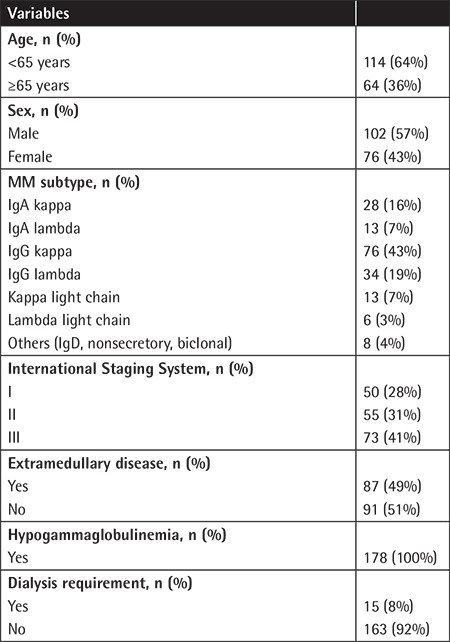
Study population characteristics.

**Table 2:**
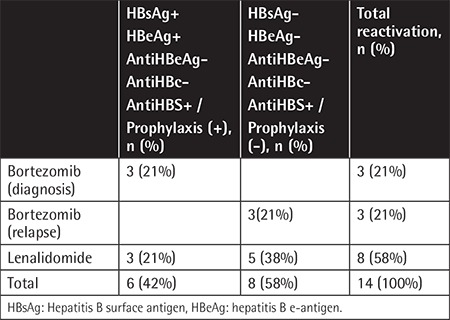
HBV and prophylaxis status at diagnosis of patients with reactivation.

**Table 3:**
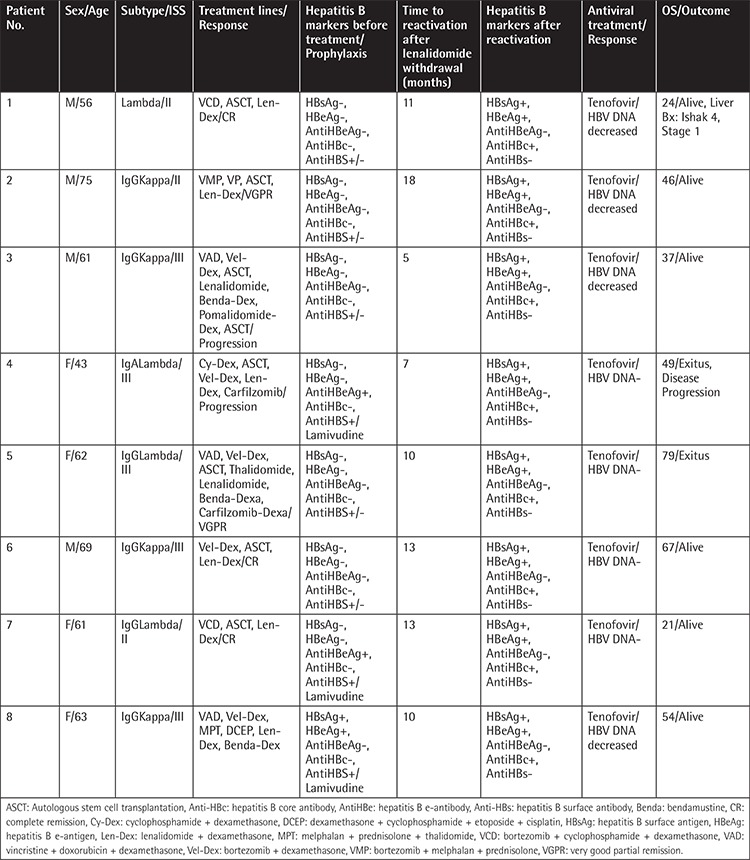
Patients with lenalidomide-related HBV reactivation.

**Table 4:**
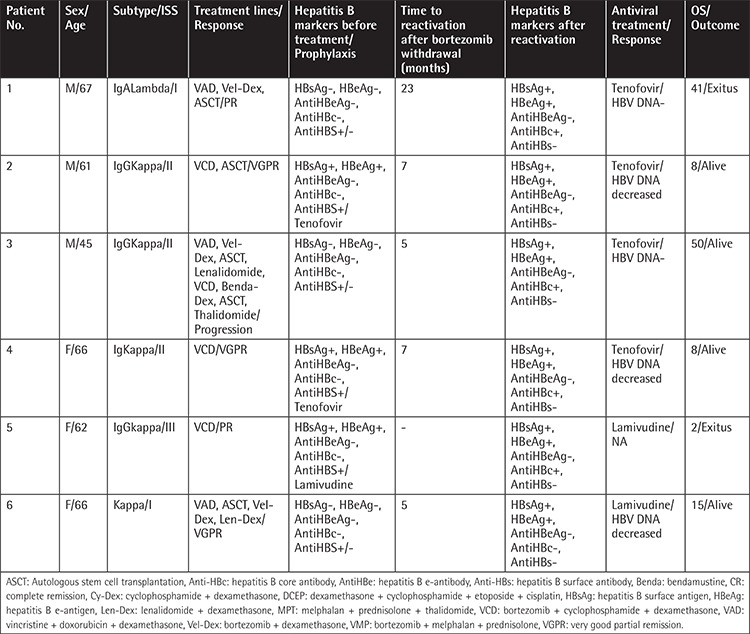
Patients with bortezomib-related HBV reactivation.
